# Identification of a Bioactive Compound against Adult T-cell Leukaemia from Bitter Gourd Seeds

**DOI:** 10.3390/plants3010018

**Published:** 2013-12-27

**Authors:** Hisahiro Kai, Ena Akamatsu, Eri Torii, Hiroko Kodama, Chizuko Yukizaki, Isao Akagi, Hisatoshi Ino, Yoichi Sakakibara, Masahito Suiko, Ikuo Yamamoto, Akihiko Okayama, Kazuhiro Morishita, Hiroaki Kataoka, Koji Matsuno

**Affiliations:** 1 Department of Pharmaceutical Health Sciences, School of Pharmaceutical Sciences, Kyushu University of Health and Welfare, 1714-1 Yoshino-machi, Nobeoka, Miyazaki 882-8508, Japan; E-Mail: kjmtsn@phoenix.ac.jp; 2 Research Promotion Bureau for Collaboration of Regional Entities, Miyazaki Prefectural Industrial Support Foundation, 16500-2 Higashi-Kaminaka, Sadowara-cho, Miyazaki, Miyazaki 880-0303, Japan; E-Mails: rojopino@mac.com (E.A.); ushio16@fc.miyazaki-u.ac.jp (E.T.); hiroyoko33@hotmail.com (H.K.); akagi046@chem.agri.kagoshima-u.ac.jp (I.A.); 3 Miyazaki Prefectural Food Research and Development Center, 16500-2 Higashi-Kaminaka, Sadowara-cho, Miyazaki, Miyazaki 880-0303, Japan; E-Mail: yukizaki@iri.pref.miyazaki.jp; 4 Department of Biochemical Science and Technology, Faculty of Agriculture, Kagoshima University, 1-21-24 Korimoto, Kagoshima, Kagoshima 890-0065, Japan; 5 Miyazaki Agricultural Experiment Station, 5805 Shimonaka, Sadowara-cho, Miyazaki, Miyazaki 880-0212, Japan; E-Mail: ino-hisatoshi@pref.miyazaki.lg.jp; 6 Department of Biochemistry and Applied Biosciences, Faculty of Agriculture, University of Miyazaki, 1-1 Gakuenkibanadai-nishi, Miyazaki, Miyazaki 889-2192, Japan; E-Mails: ysakaki@cc.miyazaki-u.ac.jp (Y.S.); msuiko@cc.miyazaki-u.ac.jp (M.S.); 7 Department of Rheumatology, Infectious Diseases and Laboratory Medicine, Faculty of Medicine, University of Miyazaki, 5200 Kihara Kiyotake, Miyazaki, Miyazaki 889-1692, Japan; E-Mails: yamamoto@fc.miyazaki-u.ac.jp (I.Y.); okayama@med.miyazaki-u.ac.jp (A.O.); 8 Division of Tumor and Cellular Biochemistry, Department of Medical Sciences, Faculty of Medicine, University of Miyazaki, 5200 Kihara Kiyotake, Miyazaki, Miyazaki 889-1692, Japan; E-Mail: kmorishi@fc.miyazaki-u.ac.jp; 9 Section of Oncopathology and Regenerative Biology, Department of Pathology, Faculty of Medicine, University of Miyazaki, 5200 Kihara Kiyotake, Miyazaki, Miyazaki 889-1692, Japan; E-Mail: mejina@med.miyazaki-u.ac.jp

**Keywords:** adult T-cell leukemia, bitter gourd seed extract, α-eleostearic acid, phytohemagglutinin-activated human peripheral blood mononuclear cell

## Abstract

In our previous report, an 80% ethanol bitter gourd seed extract (BGSE) was found to suppress proliferation of adult T-cell leukemia (ATL) cell lines. The present study aimed to identify the bioactive compounds from BGSE specific against ATL. From the result of an HPLC-MS analysis, α-eleostearic acid (α-ESA) was present in BGSE at 0.68% ± 0.0022% (±SD, *n* = 5). In the cell proliferation test, α-ESA potently suppressed proliferation of two ATL cell lines (ED and Su9T01; IC_50_ = 8.9 and 29.3 µM, respectively) more than several other octadecanoic acids. However, α-ESA moderately inhibited phytohemagglutinin-activated human peripheral blood mononuclear cells (PBMC; IC_50_ = 31.0 µM). These results suggest that BGSE-derived α-ESA has potential as a functional food constituent because of its activity against ATL, particularly against ED cells. Moreover, α-ESA might be effective for the prevention of moderate adverse effects of ATL on normal T cells.

## 1. Introduction

Adult T-cell leukemia (ATL) occurs in a small population of human T-cell leukemia virus type I (HTLV-I) infected individuals. After transmission of HTLV-I, 2%–5% of carriers are likely to develop ATL after a long latency period (30–50 years) [1]. These patients have been frequently identified as being from a restricted area of tropical regions [2]. It is currently very difficult to effectively treat patients with ATL using existing therapeutic methods, and most clinical trials focus on chemotherapeutic treatment and allogeneic hematopoietic stem cell transplantation. Therefore, it is important to find appropriate therapeutic methods to prevent the development of ATL or to prolong survival after its occurrence.

In our previous report, we screened 52 agricultural plant samples for their ability to inhibit proliferation in seven kinds of ATL related cell lines to start structure of a study for finding potential drug candidates with the prevention of ATL. We found that an 80% ethanol bitter gourd (*Momordica charantia* L.) seed extract (BGSE) showed an inhibitory effect on the proliferation of ATL-related human leukemia cells [3].

Bitter gourd belongs to the Cucurbitaceae family and is cultivated worldwide as a vegetable crop. The fruit is not only used as a food, but also for its medicinal properties, such as anti-microbial, anti-diabetic, anti-HIV and anti-tumor activities, which were described in a recent review [4]. BGSE has also been reported to have anti-leukemic potential on human acute myelogenous leukemia cells (HL-60) [5]. However, HL-60 and ATL cell lines comprise different types of leukemia cells, and as there have been no reports about bioactive compounds from BGSE active against ATL, the effect of BGSE on ATL cell proliferation requires elucidation. The aim of the present study was to identify bioactive compounds in BGSE exhibiting activity against ATL.

## 2. Results and Discussion

### 2.1. Identification of Active Compounds in BGSE

**Figure 1:**
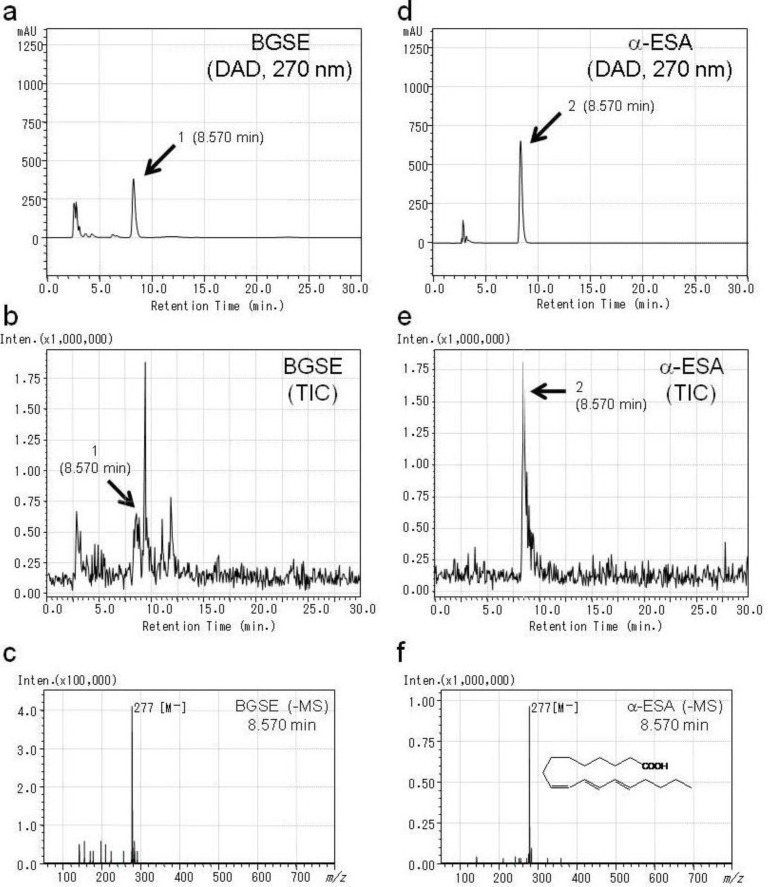
HPLC-DAD and MS chromatograms of BGSE and α-ESA. (**a**,**d**) HPLC-DAD chromatograms (270 nm) of BGSE (**a**) and α-ESA (**d**). (**b**,**e**) Total ion chromatograms (TIC) of BGSE (**b**) and α-ESA (**e**). (**c**) MS spectrum (negative-ion spectra) of peak 1 of BGSE in Figure 1b. (**f**) MS spectrum (negative-ion spectra) of peak 2 of α-ESA in Figure 1e.

Figure 1a,b show the HPLC-DAD chromatogram (a) and HPLC-MS-total ion chromatogram (TIC) (b) of BGSE. As shown in Figure 1a, a major peak (peak 1: retention time (RT) = 8.570 min, λ = 270 nm) was detected for BGSE. The TIC showed several peaks (Figure 1b). Figure 1c shows the MS spectrum of the peak with the RT: 8.570 min. in Figure 1b, which shows a deprotonated molecular ion signal at *m*/*z* 277. This compound was estimated to be C_18_H_29_O_2_ and was proposed to be α-ESA on the basis of reference data [6]. α-ESA was analyzed using the same method. Figure 1d–f shows the HPLC-DAD chromatogram (d), TIC (e) and MS spectrum (f) of α-ESA. α-ESA (50 µg/mL) showed a clear peak at 270 nm (Figure 1d, peak 2) and the same RT of peak 1 in Figure 1a (BGSE). The RT (8.570 min.) of the highest peak in TIC for α-ESA (Figure 1e, peak 2) was the same as peak 1 in Figure 1b. The MS spectrum of α-ESA (Figure 1f) showed the same result as the analysis of BGSE (Figure 1c). From these results, peak 1 in Figure 1 was determined to be α-ESA, and α-ESA is the main compound in BGSE.

**Figure 2:**
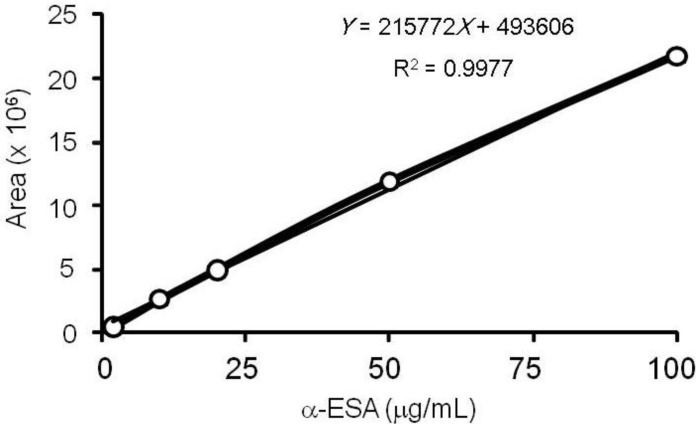
Calibration curve of α-ESA.

We also attempted to quantitatively determine α-ESA in BGSE. Figure 2 shows the α-ESA calibration curve. The calibration curve showed good linearity (R^2^ = 0.9977). α-ESA was contained in the concentration range of 34.0 and 34.2 µg/5 mg of freeze-dried bitter gourd seeds (0.68% ± 0.0022%, ±SD, *n* = 5). In our previous report, BGSE suppressed the proliferation of ATL cell lines [3]. The current study shows the presence of α-ESA in BGSE. Tsuzuki *et al*. reported that α-ESA accounted for about 60% of the total fatty acid composition of bitter gourd seed oil [7]. Therefore, α-ESA might have greatly contributed to suppressing the proliferation of ATL cell lines.

### 2.2. The Inhibitory Effects of Octadecanoic Acid Analogs on ATL Cell Lines

Of the octadecanoic acids, conjugated linoleic acids (CLA), which includes α-ESA, has been acknowledged to have numerous biological activities, such as anti-obesity, anti-diabetes, anti-cancer, anti-arthritis, anti-asthma, and anti-cardiovascular disease effects [8]. However, no study has yet reported the anti-leukemic effects of α-ESA on ATL cell lines. We examined the inhibitory effects of related compounds, seven octadecanoic acid groups, on the proliferation of two types of ATL cell lines (ED and Su9T01). As shown in Table 1, α-ESA (C18:3, n-5), γ-linolenic acid (C18:3, n-6), and α-linolenic acid (C18:3, n-3), which belong to the triunsaturated fatty acid group, substantially inhibited ED cell growth (IC_50_ values of 8.9, 61.3 and 129.9 µM, respectively) and Su9T01 cell growth (IC_50_ values of 29.3, 174.3 and 167.4 µM, respectively). Linoleic acid (C18:2, n-6), which belongs to the diunsaturated fatty acid group, inhibited ED and Su9T01 cell growth (IC_50_ values of 100.7 and 180.1 µM, respectively). In ED cells, these compounds exhibited higher activity than EGCG, which was used as the positive control [9] (IC_50_ = 152.7 µM). On the other hand, in Su9T01 cells, only α-ESA exhibited higher inhibitory activity than EGCG (IC_50_ = 166.0 µM). We cannot calculate the exactly IC_50_ values of monounsaturated fatty acid group (oleic acid (C18:1, n-9) and elaidic acid (C18:1, n-9)) and saturated fatty acid (stearic acid (C18:0)) in both cell lines. This assay was employed to compare the effects of octadecanoic acids on ED and Su9T01 cell growth; α-ESA showed the highest inhibitory activity. IC_50_ values decreased roughly in proportion to the number of double bonds; therefore, the number of double bonds was an important determinant of anti-proliferation activity. Indeed, the triunsaturated fatty acid group was more potent than EGCG.

**Table 1:** Relationship between octadecanoic acid structure and inhibition of adult T-cell leukemia (ATL) cell line proliferation.

Compounds		IC_50_ (µM)	
		ED	Su9T01
α-ESA	(C18:3, n-5)	8.9	29.3
γ-linolenic acid	(C18:3, n-6)	61.3	174.3
α-linolenic acid	(C18:3, n-3)	129.9	167.4
linoleic acid	(C18:2, n-6)	100.7	180.1
oleic acid	(C18:1, n-9)	500.0–166.7	500.0–166.7
elaidic acid	(C18:1, n-9)	>500.0	>500.0
stearic acid	(C18:0)	500.0–166.7	>500.0
EGCG	(Positive Control)	152.7	166.0

ATL cells (ED and Su9T01) were incubated for 72 h in RPMI-1640 medium containing each compound. Viable cells were detected using a WST-8 assay kit. The concentration at which cell proliferation is inhibited by 50% compared to untreated control is expressed as IC_50_.

### 2.3. The Effect of α-ESA on ATL Cell Line and Phytohemagglutinin-Activated Human Peripheral Blood Mononuclear Cell (PBMC) Proliferation

**Figure 3:**
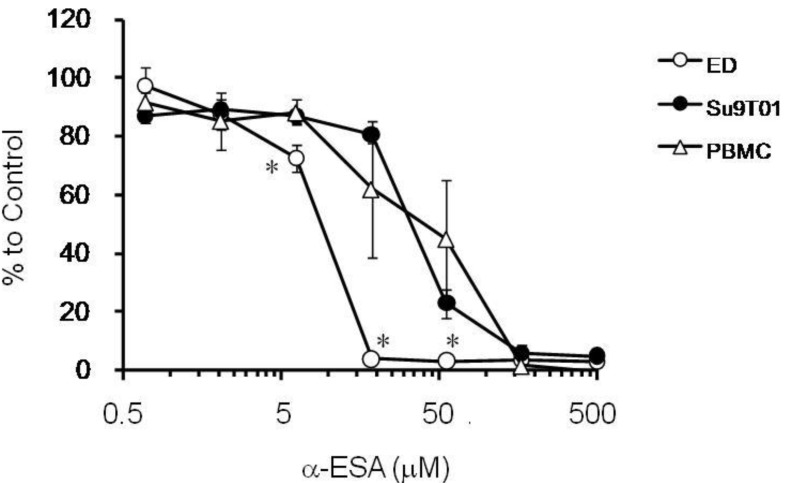
Effect of α-ESA on ATL cell and PBMC proliferation.

As shown in Figure 3, we compared the suppressive effect of α-ESA on ED, Su9T01 cells and PBMCs. PBMCs are commonly used as the healthy/normal cell model in comparison to cancer cell lines. Significant differences were observed between ED cells and PBMCs treated at 6, 19 and 56 µM α-ESA (*p* < 0.05). Su9T01 cells and PBMCs treated with between 1 and 500 µM α-ESA were not significantly different. These results confirmed that the α-ESA strongly and selectively inhibited ED cells and moderately inhibited PBMCs, which are healthy normal T cells. α-ESA at 166 and 500 µM significantly decreased proliferation of all cell types. α-ESA showed inhibitory effects on ED, Su9T01 cells and PBMCs (IC_50_ of 8.9, 29.3 and 31.0 µM, respectively).

A comparison of the dose-dependent effects of α-ESA on ED, Su9T01 cells and PBMCs. Each mark represents the mean ± SD of three independent tests (Student’s *t*-test). Significantly different between ED cells and PBMCs: *p* < 0.05 (*).

Tsuzuki *et al*. and Kobori *et al*. reported that bitter gourd seed oil and its constituent α-ESA had anti-leukemic potential in HL-60 cells [5,10]. While HL-60 and ATL (ED and Su9T01) are leukemia cell lines, they differ in origin and whether they are a result of viral infection. The antiproliferative properties of unsaturated fatty acids are well known. For example, Wendel *et al*. reported that the unsaturated fatty acids (mainly omega-3 fatty acids and derivatives) like conjugated eicosapentaenoic acid as important nutritional adjuvant therapeutics in the management of various human cancer diseases and the impact of nutritional omega-3 fatty acids on cancer prevention [11]. α-ESA also may have similar potential with nutritional function for cancer prevention. The present study is the first to show the inhibitory effects of α-ESA on ATL cells *in vitro*. Specifically, α-ESA showed an inhibitory effect in the rank order: ED cells > Su9T01 cells ≥ PBMCs. Sasaki *et al*. reported that tumor suppressor in lung cancer 1 (*TSLC1*) gene expression was different between ED and Su9T01 cells [12,13]. Therefore, using gene expression analysis, future studies should investigate the regulatory mechanism of *TSLC1* and its DNA methylation, as well as the possible role of α-ESA in the inhibition of ED and Su9T01 proliferation and *TSLC1* expression.

## 3. Experimental

### 3.1. Chemicals

α-Eleostearic acid (α-ESA) was obtained from Larodan Fine Chemicals AB, Malmö, Sweden and Cayman Chemical, Ann Arbor, MI, USA. γ-Linolenic acid, linoleic acid, α-linolenic acid, and elaidic acid were purchased from Cayman Chemical. Stearic acid and oleic acid were purchased from Wako, Osaka, Japan. Epigallocatechin-3-gallate (EGCG) was purchased from Nagara Science Co., Gifu, Japan. Ficoll was purchased from GE Healthcare, Uppsala, Sweden. Phytohemagglutinin (M Form) was purchased from Invitrogen, Carlsbad, CA, USA. IL-2 was purchased from R&D Systems, Minneapolis, MN, USA. A 2-(2-methoxy-4-nitrophenyl)-3-(4-nitrophenyl)-5-(2,4-disulfophenyl)-2*H*-tetrazolium monosodium salt (WST-8) assay kit was purchased from Dojindo, Kumamoto, Japan.

### 3.2. Identification of Compounds in BGSE

The freeze-dried powder of bitter gourd seeds (5 mg) was extracted with 80% EtOH (0.5 mL) by vortexing for 30 s, followed by centrifugation at 1,500 rpm for 3 min. The supernatant was used for high performance liquid chromatography-diode array detector (HPLC-DAD) and mass spectrometry (MS) analysis. The α-ESA HPLC analysis method was a modification of the methods of Amakura *et al*. and Řezanka *et al*. [6,14]. The HPLC-DAD and MS analysis consisted of a Shimadzu HPLC System (LC-20A Prominence, Shimadzu, Kyoto, Japan) coupled to a SPD-20A (DAD; Shimadzu, Kyoto, Japan) and an LC/MS-ion trap-time of flight (LC/MS-IT-TOF, Shimadzu, Kyoto, Japan) fitted with an atmospheric pressure chemical ionization (APCI) source. HPLC separation was performed on a reverse-phase column (Atlantis T3, 2.1 mm I.D. ϕ100 mm, 3 μm; Waters, Milford, MA, USA). The column was maintained at 40 °C. The mobile phase consisted of eluent A (0.1% acetic acid and MeOH)/eluent B (0.1% acetic acid and 10% MeOH aq.) = 90:10 at a flow rate of 0.10 mL/min. The injection volume was 10 μL. APCI conditions were recorded from *m*/*z* = 50 to 400 in negative ion mode. The other MS conditions were as follows: nebulizer N_2_ gas, 2.5 L/min; APCI interface temperature, 400.0 °C; curved desolvation line (CDL) temperature, 250.0 °C; heat block temperature, 200.0 °C; detector voltage, 1.80 kV.

### 3.3. α-ESA Calibration Curve

The α-ESA standard was dissolved in 80% ethanol and serial dilutions were analyzed by HPLC-DAD. α-ESA content was calculated using the following linear equation based on the calibration curve: *Y* = 215772*X* + 493606, R^2^ = 0.9977. *Y* is the area detected by DAD (270 nm), and *X* is the α-ESA content in μg/mL.

### 3.4. ATL Cell Proliferation Assay

We used two ATL cell lines (ED and Su9T01) that are highly sensitive to inhibition of cell proliferation, as determined in our previous study [3]. ED cells were kindly provided by Dr. M. Maeda (Kyoto University, Kyoto, Japan) and Su9T01 cells were kindly provided by Dr. N. Arima (Kagoshima University, Kagoshima, Japan). The test compounds were dissolved in dimethyl sulfoxide and subjected to assay screening. The method of ATL assay is described in a previous report [3]. IC_50_ calculation was some curve fitted onto the determined proliferation inhibition points.

### 3.5. Isolation and Culture of PBMCs

The method of isolation and culture of PBMCs is as follows. Heparinised blood (5 mL) was diluted by adding 5 mL of PBS. The diluted blood samples were divided into four equal parts, loaded on 4 mL of Ficoll and centrifuged at 400 × *g* for 30 min. The PBMC layer was located within the interphase between the Ficoll and plasma. The Ficoll contained the erythrocytes and most of the granulocytes. The plasma was removed using a pipette until ~5 mL above the PBMC interphase. The cells were washed three times with PBS (centrifuged at 200 × *g* for 15 min) and resuspended in RPMI 1640 medium supplemented with 10% foetal bovine serum containing 100 U/mL penicillin G, 100 μg/mL streptomycin, 2 ng/mL IL-2 and 128-fold dilution of phytohemagglutinin to a final cell density of 1 × 10^6^ cells/mL. The PBMC proliferation assay was conducted using the same method as for the ATL proliferation assay [3].

### 3.6. Statistics

Each experiment was conducted at least three times. All data are expressed as the mean ± standard deviation (SD) of three independent experiments. Statistically significant differences were calculated by Student’s *t*-test.

## 4. Conclusions

α-ESA was shown to be the main bioactive compound in BGSE, and contributes to the inhibition of ED cell differentiation and proliferation without damaging normal cells, leading to the disruption of ATL pathogenesis.
